# Association of IL-10 gene polymorphisms and susceptibility to Juvenile Idiopathic Arthritis in Egyptian children and adolescents: a case-control study

**DOI:** 10.1186/s13052-017-0328-1

**Published:** 2017-01-17

**Authors:** Manar M. Fathy, Hosam F. Elsaadany, Yasser F. Ali, Mohsen A. A. Farghaly, Mohammed E. Hamed, Hany E. Ibrahim, Maha A. Noah, Mayy A. N. Allah, Shaimaa S. A. Elashkar, Nasser I. Abdelsalam, Hind M. Abdelrahman, Ahmed R. Ahmed, Heba G. Anany, Sanaa M. Ismail, Boshra R. Ibrahim, Nashwa M. Al Azizi, Heba H. Gawish, Ghada M. Al-Akad, Rehab M. Nabil, Dalia S. Fahmy, Salah F. Alsayed

**Affiliations:** 1 Department of Pediatrics, Faculty of Medicine, Zagazig University, 13 Omar Bin Elkhattab St, Al Qawmia, Zagazig City, AlSharqia Governorate Egypt; 2 Department of Pediatrics, Faculty of Medicine, Aswan University, Aswan, Egypt; 3 Department of Clinical Pathology, Faculty of Medicine, Zagazig University, Zagazig, Egypt; 4 Department of Rheumatology, Faculty of Medicine, Zagazig University, Zagazig, Egypt; 5 Department of Internal Medicine, Faculty of Medicine, Zagazig University, Zagazig, Egypt

**Keywords:** Juvenile idiopathic arthritis, Gene polymorphisms, Cytokines, Interleukin-10

## Abstract

**Background:**

Juvenile Idiopathic Arthritis (JIA) is the most common chronic arthritis in children worldwide. Among anti-inflammatory cytokines, interleukin-10 (IL-10) is a key immunosuppressive cytokine involved in the pathogenesis of JIA. To date, only a few studies concerned the association of interleukin-10 gene polymorphisms with JIA. In this study, we aimed to investigate 3 cytokine single-nucleotide polymorphisms situated at positions -1082(G/A), −819(C/T), and −592(C/A) in the promoter region of the IL-10 gene to determine whether this polymorphism could be a marker of susceptibility to JIA in Egyptian children and adolescents. We also measured the serum level of IL-10 to assess its relation to such polymorphism.

**Methods:**

This was a case-control study included 100 patients diagnosed with JIA, and matched with age, gender, ethnicity 100 healthy control subjects.

Interleukin-10 −1082(G/A), −819(C/T), and −592(C/A) polymorphisms were genotyped by amplification refractory mutation system- polymerase chain reaction (ARMS)-PCR methodology, while the serum IL10 levels were measured by *ELISA* method.

**Results:**

Compared to the controls subjects, the frequency of IL-10- AA genotype and A allele at the –1082 position were overrepresented in patients with JIA (OR = 2.7; 95% CI: 1.1–6.4 for the AA genotype; *P* <0.05 and OR: 1.5; 95% CI: 1.03–2.3 for the A allele; *P* <0.05 respectively). On the other hand, no significant differences were found between the 2 groups in the genotype or allele frequencies for the –819 and –592 positions. Of note, we found a significant positive association between the IL-10 (-1082) AA genotype and susceptibility to polyarticular JIA (OR: 4.3; 95% CI: 1.5–12.7; *P* <0.01). We observed that patients with the IL-10 (-1082) AA genotype had significantly lower serum IL-10 levels (2.3 ± 0.9 *pg/ml*) compared to those with AG genotype (7.6 ± 1.5 *pg/ml*) and GG genotype (9.5 ± 1.2 *pg/ml*); *P* < 0.01, respectively.

**Conclusion:**

We demonstrate for the first time, to the best of our knowledge, that the presence of an A allele or AA gene variant at the –1082 position of the promoter region of the interleukin-10 gene may constitute risk factors for developing JIA in Egyptian children and adolescents. Moreover, we observed a significant positive association between the IL10 –1082 AA gene variant and susceptibility to polyarticular JIA.

## Background

Juvenile idiopathic arthritis (JIA) is defined as arthritis in a child under the age of 16 years affecting one or more joints, lasting for at least 6 weeks and currently having no other known etiology [1]. JIA is the most common autoimmune inflammatory joint disease, with an incidence of 2.6 to 23 cases per 100,000 children per year [2]. JIA may lead to long-term morbidities such as uveitis, depression, osteoporosis, poor pain control, and even severe disabilities due to joint damage [3]. The mechanism underlying joint damage in JIA is complex and involves a variety of molecular and cellular processes that may be influenced by genetic factors [4].

Cytokines have been implicated in the development and perpetuation of inflammatory response in JIA [5]*.* During active disease, cytokine concentrations in plasma of patients with JIA increased 2 to 35‐fold [6]. A central feature of JIA is a relative imbalance of cytokine profile, with a relative excess of pro-inflammatory molecules including interleukin-1, interleukin -6, and tumor necrosis factor (TNF-α) compared with anti-inflammatory mediators such as interleukin -10 [7]. This idea has raised a great deal of interest in the role of the immunologic system in the pathogenesis of JIA.

Interleukin-10 (IL-10) is a key immunosuppressive cytokine that is produced by a wide range of leukocytes, as well as non-hematopoietic cells [8]. An earlier study by Hart et al. reported that IL-10 can effectively block the production of the pro-inflammatory cytokines IL-1, IL-8, and TNF-α by snivel macrophages and synoviocytes. IL-10 was also correlated with an increased autoantibody production and B cell activation in RA patients [9].

Previous studies revealed increased levels in plasma and synovial fluid of some inflammatory cytokines in patients with JIA [10, 11]. Prahalad et al. [10] compared serum cytokine levels between 77 children with different JIA subtypes and 81 age-matched healthy controls. The authors reported that IL10 was significantly elevated in patients with polyarticular as well as systemic JIA. This research added that IL-6 and TNF-α were positively associated with JIA, while IL10 might have a protective effect on disease, as might be anticipated by its inhibitory activity.

Despite these studies, the association of interleukin-10 levels with JIA does not constitute proof of cause. One way to clarify the issue of causation involves a genetic approach. The IL-10 gene has been mapped to chromosome 1q31-32, and three single nucleotide polymorphisms (SNPs) in the promoter region at positions -1082(G/A), −819(C/T), and −592(C/A) have been described [12]. Polymorphisms within genes encoding inflammatory cytokines are known to alter the production of cytokines [13]. To date, only a few studies concerned the association of interleukin-10 gene polymorphisms with JIA and the susceptibility to JIA.

On the basis of these considerations, we designed this study to investigate 3 cytokine single-nucleotide polymorphisms situated at positions −1082(G/A), −819(C/T), and −592(C/A) in the promoter region of the interleukin-10 gene to determine whether this polymorphism could be a marker of susceptibility to JIA in Egyptian children and adolescents. We also measured the serum level of IL-10 to assess its relation to such polymorphism.

## Methods

This was a prospective case-control study performed in Zagazig University Hospitals, and outpatient clinics in the same hospitals from April 2014 to October 2016. This study was approved by the ethical committee of Zagazig University, Egypt and written informed consent from parents (and where appropriate patients) was provided in accordance with the Declaration of Helsinki.

One hundred children and adolescents; who had JIA as diagnosed in the Department of Pediatrics in the same hospital, were enrolled in this study. The age of the patients ranged from 9 to 16 years (median, 11 years). Diagnosis of JIA followed the criteria established in the International League of Associations for Rheumatology (ILAR) [14]. Patients were divided into three major subgroups: oligoarticular JIA, polyarticular JIA (including five extended oligoarticular JIA) and systemic JIA. All patients had active disease. Active disease was defined by the presence of joint swelling or limitation of movement with either pain on movement or tenderness [15]. In systemic JIA, fever and laboratory evaluation of erythrocyte sedimentation rate (ESR) and C‐reactive protein (CRP) levels were used as additional parameters for disease activity. All patients were subjected to proper history taking placing special focus on number of affected joints, and medications used to treat JIA at the time of enrollment; thorough clinical examination and radiographs were performed to the hands and feet to detect any erosions or joint space narrowing.

One hundred healthy children and adolescents, of comparable age and gender; who attended Pediatric Department for preoperative evaluation for elective surgery, were enrolled as control group. Patients and controls belonged to the same ethnic group: African Caucasian.

### Blood sampling

Blood samples were drawn from all subjects at admission and divided into two portions: 2 ml of whole blood was collected into tubes containing EDTA, for genomic DNA extraction. Serum were separated immediately from remaining part of the sample and stored at −20 °C till the time of analysis.

### Genomic DNA extraction

Genomic DNA from venous blood samples of JIA patients and healthy controls were extracted using a genomic DNA extraction kit (Puregene Blood Kit, Gentra, Valencia, USA) according to the manufacturer’s protocol. DNA quantification was done using an Eppendorf Bio Photometer (New York, USA). DNA was stored at −20 °C.

### IL10 genotyping

All subjects were genotyped for IL10- polymorphism by amplification refractory mutation system (ARMS)-PCR methodology. For the polymorphism at position – 1082 of interleukin-10, a 238 base-pair region was amplified by using the sense primers 5'TTCCCCAGGTAGAGCAACACT-3' and the antisense primer 5'GATGGGGTGGAAGAAGTTGAA-3'. For the polymorphisms at positions −819 and −592 of interleukin-10, a 467 base-pair region was amplified using the sense primer 5'AACTTCTTCCACCCCATCTTT-3' and antisense primer 5'-ATCCTCAAAGTTCCCAAGCAG-3'. as described before [16]. A 25-μl PCR reaction mixture contained 10 ng of genomic DNA, 10 pmol of each of 5' -or 3' - primer, 100 μmol/L dNTP, 50 mmol/L KCL, 10 mmol/L Tris-HCl (pH 8.3) 1.5 mmol/L Mgcl2 and 1 U of AmpliTaq polymerase. Amplifications were performed using a Perkin-Elmer 2400 thermal cycler according the following parameters: 95 °C for 3 min followed by 29 cycles of 95 °C for 30 s, 64 °C for 20 s, and 72 °C for 30 s. A final extension at 72 °C was performed for 10 min. PCR products were digested overnight at 37 °C using 2.5 U of restriction enzyme. The digested products were analyzed on a 2% agarose gel stained with ethidium bromide.

### Measurement of serum interleukin-10 (IL-10) levels

IL-10 plasma levels were measured using an enzyme-linked immunosorbent assay (*ELISA*; CLB, Pelikine Compact human IL-10 *ELISA* kit, Amsterdam, The Netherlands). The sensitivity of the assay was 1 pg/ml and the assay was performed according to the manufacturer’s instructions. The manufacturer reports an intra-assay coefficient of variation of less than 10% and an inter-assay coefficient of variation of less than 10%.

### Statistical analysis

IL-10 genotype and allele frequencies in patients and controls were tested for Hardy-Weinberg equilibrium. Chi-square test was used to determine differences in the frequencies of the different IL-10 genotypes between patients and controls and between clinical subtypes within JIA patients. The odds ratio (OR) and 95% confidence interval (95% CI) were calculated for disease susceptibility and clinical subtypes in relation to the studied IL-10 (SNP) gene polymorphisms. The Student t test and analysis of variance were used to compare numeric variables within groups, depending on the distribution of the data. *P* value < 0.05 was considered to be statistically significant. All data were analyzed using the *Epi Info* statistical software (version 6.2, World Health Organization, Geneva, Switzerland).

## Results

Our study included 100 patients with JIA, their age ranged from 9 to 16 years (median 11 years), 35 males and 65 females and 100 healthy control subjects whose clinical characteristics are listed in Table 1. The control group were age and gender matched to patients with JIA. The mean age at onset of JIA in the patients was 9.7 years and mean disease duration was 3.5 years.

**Table 1:** Baseline clinical and laboratory data of patients with Juvenile idiopathic arthritis (JIA) and control group

	Patients *n* = 100	Controls *n* = 100	*P*
*Age, years*	7.5 (5–16)	7.7 (5–16)	>0.05^a^
*Gender (M/F)*	35/65	39/61	>0.05^b^
*Subtype of JIA :*			
Systemic	23 (23%)	-	-
Polyarticular	31 (31%)	-	-
Oligoarticular	46 (46%)		
*Age at onset of JIA (years)*	6.7 ± 1.4	-	-
*Medications used on admission*			
NSAIDS % alone	16 (16%)	-	-
NSAIDS, MTX	29 (29%)	-	-
NSAIDS, steroids	28 (28%)		
NSAIDS, MTX, steroids	24 (24%)		
NSAIDS, MTX, biologic	3 (3%)		

Abbreviations: *IL10* interleukin 10

Data are presented as median (Range) or mean ± SD

*P* value < 0.05 indicates a significant difference

^a^Mann-Whitney U test. ^b^Chi-square test

Of these patients, 23 (23%) had systemic JIA, 31(31%) had polyarticular JIA (nine patients were RF positive), and 46(46%) had oligoarticular JIA. On admission, 16% of JIA patients were receiving a non-steroidal anti-inflammatory agent (NSAIDS), 29% were on NSAIDS and methotrexate(MTX), 28% were on NSAIDS and steroids,24% were on triple therapy of NSAIDS, MTX, steroids, and 3% were on NSAIDS, MTX, biologic agents (Table 1).

Distribution of IL-10 genotypes, alleles and serum IL-10 levels in patients with JIA and controls are summarized in Table 2. Both groups were in Hardy-Weinberg equilibrium, with no significant chi-squared values for the observed and expected genotype frequencies.

**Table 2:** Distribution of IL-10 genotypes, alleles and serum IL-10 levels in patients with Juvenile idiopathic arthritis (JIA) and control group

Genotype		Patient group		Control group		*OR *(*95% CI*)	*P*
		n (100)	%	n (100)	%		
IL-10 (-1082)	AA	23	(23)	10	(10)	2.7 (1.1–6.4)	<0.05
	AG	69	(69)	73	(73)		
	GG	8	(8)	17	(17)		
Alleles							
	A	115	(57.5)	93	(46.5)	1.5 (1.03–2.3)	< 0.05
	G	85	(42.5)	107	(53.5)	0.6 (0.4–0.9)	
−819/-592	TT/AA	23	(23)	19	(19)		> 0.05
	CT/CA	65	(65)	71	(71)		
	CC/CC	12	(12)	10	(10)		
Alleles							
	T/A	111	(55.5)	109	(54.5)		> 0.05
	C/C	89	(44.5)	91	(45.5)		
Serum IL10 (pg/ml)		7.4 ± 1.5		5.6 ± 1.3			> 0.05^a^

Abbreviations: *IL-10* interleukin-10, *OR* odds ratio, *CI* 95% confidence interval

Values in parentheses are percentages or data are presented as mean ± SD

*P* value < 0.05 indicates a significant difference. Chi-square test. ^a^ *Student t-test*

The IL-10-1082 genotype distribution differed between patients with JIA and healthy controls. The AA homozygous genotype was overrepresented (23%) among JIA patients, compared with controls (10%). Homozygous subjects had a 2.7 fold increased risk of developing JIA (OR = 2.7; 95% CI: 1.1–6.4; *P* <0.05), while genotypes AG/GG were not representative for JIA patients (OR = 0.37; 95% CI: 0.15–0.88; *P* <0.05); Table 2.

Of note, we found a significant increase in the frequency of the A allele (57.5%, OR: 1.5; 95% CI: 1.03–2.3; *P* <0.05) at the −1082 position in IL-10 gene among JIA patients; where a concomitant significant decrease in the frequency of the G allele at the same position was observed compared to the control group (42.5%, OR: 0.6; 95% CI: 0.4–0.9; *P* <0.05); Table 2.

On the other hand, no significant differences were found between the 2 groups in the genotype or allele frequencies for the −819 and −592 positions, which shows linkage disequilibrium; (*P* >0.05), Table 2. Complete linkage disequilibrium was observed between the −819 and −592 alleles. All the samples homozygous for allele C at −819 were homozygous for allele C at −592, and all the samples homozygous for allele T at −819 were homozygous for A at −592; furthermore, heterozygote samples at position −819 were invariably heterozygous at −592. Therefore, −819 and −592 genotypes and alleles occurred at identical frequencies (Table 2).

We therefore examined the IL-10 genotypes of the JIA patients with regard to clinical and laboratory parameters. No significant differences were observed when we analyzed IL-10 genotypes according to gender, presence of rheumatoid factor, extra-articular manifestations or mean disease duration.

Our data revealed that patients with JIA had serum IL-10 levels similar to the control group (7.4 ± 1.5 pg/ml vs 5.6 ± 1.3 pg/ml; *P* >0.05); Table 2.

Interestingly, when our patients were evaluated for their clinical subtypes of JIA, there was a significant positive association between the IL-10 (-1082) AA genotype and susceptibility to polyarticular JIA (OR: 4.3; 95% CI: 1.5–12.7 for the AA genotype; *P* <0.01); Table 3. On the other hand, no significant association was evident between genotype or allele frequencies for the −819 and −592 positions and different clinical subtypes of JIA among studied patients; Table 3.

**Table 3:** Comparison of Genotype and Allele Frequency of IL-10 Polymorphisms between different clinical subtypes of JIA among studied patients

Genotype		sJIA	polyJIA	oligoJIA	*OR *(*95% CI*)	*P*
		*n* = 23 (%)	*n* = 31 (%)	*n* = 46 (%)		
IL-10 (-1082)	AA	6 (26)	13 (42) *	4 (9)	4.3 (1.5–12.7)	< 0.01
	AG	16 (70)	15 (48)	38 (82)		
	GG	1 (4)	3 (10)	4 (9)		
Alleles						
	A	28 (61)	41 (66)	46 (50)		> 0.05
	G	18 (39)	21 (34)	46 (50)		
−819/−592	TT/AA	5 (22)	8 (26)	10 (22)		> 0.05
	CT/CA	14 (61)	17 (55)	34 (74)		
	CC/CC	4 (17)	6 (19)	2 (4)		
Alleles						
	T/A	24 (55)	33 (54)	54 (17)		> 0.05
	C/C	22 (44)	29 (45)	38 (17)		
Serum IL10 (pg/ml)		5.7 ± 1.5	2.5 ± 1.3	9.6 ± 1.7		< 0.01^a^

Abbreviations: *IL-10*, interleukin-10, *sJIA* systemic Juvenile idiopathic arthritis, *poly JIA* polyarticular Juvenile idiopathic arthritis, *oligoJIA* oligoarticular Juvenile idiopathic arthritis, *OR* odds ratio, *CI* 95% confidence interval

Values in parentheses are percentages or data are presented as mean ± SD

*P value < 0.05 indicates a significant difference. Chi-square test. ^a^ *ANOVA-test*

We observed that patients with polyarticular JIA did show significantly lower serum IL-10 levels (2.5 ± 1.3 *pg/ml*) compared to those with systemic JIA (5.7 ± 1.5 *pg/ml*) and patients with oligoarticular JIA (9.6 ± 1.7 *pg/ml*); *P* <0.01, Table 3.

Of note, we observed that patients with the IL-10 (-1082) AA genotype had significantly lower serum IL-10 levels (2.3 ± 0.9 *pg/ml*) compared to those with AG genotype (7.6 ± 1.5 *pg/ml*) and GG genotype (9.5 ± 1.2 *pg/ml*); *P* < 0.01, respectively. The mean serum IL-10 level was 3.5 ± 0.8 *pg/ml* for the A allele vs 10.7 ± 1.4 *pg/ml* for the G allele; *P* < 0.01, Table 4. However, we could not find any significant association between IL-10 genotype or allele frequencies and serum IL-10 levels for the −819 and −592 positions in studied patients with JIA (*P* > 0.05); Table 4.

**Table 4:** Association of IL-10 genotypes and alleles with serum IL-10 levels in patients with JIA

*Gene polymorphism*	*Genotype/Alleles*	*Serum IL10 (pg/ml*)	*P*
IL-10 (−1082)	AA	2.3 ± 0.9	<0.01^a^
	AG	7.6 ± 1.5	
	GG	9.5 ± 1.2	
Alleles			
	A	3.5 ± 0.8	<0.01^b^
	G	10.7 ± 1.4	
IL-10 −819/−592	TT/AA	8.4 ± 1.5	>0.05^a^
	CT/CA	7.8 ± 1.7	
	CC/CC	9.7 ± 1.3	
Alleles	T/A	7.2 ± 1.3	>0.05^b^
	C/C	9.5 ± 1.6	

Abbreviations: *IL10* interleukin-10

Data are presented as mean ± SD

*P* value < 0.05 indicates a significant difference

^a^Calculated by *ANOVA* test ^b^ *Student t-test*

## Discussion

Juvenile Idiopathic Arthritis (JIA) is the most common chronic arthritis in children worldwide. Children with JIA can experience delayed and restricted growth, with an estimated 49% of affected children end up with severe functional limitations [17].

A complex interaction between individual gene susceptibility, cytokines activation, and various environmental triggers may lead to an immune imbalance that subsequently results in articular and systemic manifestations of JIA [10]. Cytokines play a pivotal role in immune response and inflammation. Among these cytokines, IL-10 is a potent pleiotropic cytokine that is produced primarily by monocytes and to a lesser extent by Th-2 lymphocytes. This cytokine has the capacity to inhibit the synthesis of pro-inflammatory cytokines such as IL-6, IL-2, IFN-γ, and TNF-α [18]. An association between the polymorphisms of IL-10 gene and various inflammatory and autoimmune diseases including Juvenile Rheumatoid Arthritis [19], Behcet’s disease, non-infectious uveitis, and Type 1 diabetes have been reported [20, 21].

Because of potential immune-modulatory effects of IL-10 and its importance as a major anti-inflammatory cytokine, IL-10 gene polymorphisms might affect individual susceptibility to JIA.

In the present study, we found that IL-10- AA gene variant and A allele at the –1082 position were overrepresented in patients with JIA compared to the control group. In addition, we observed that individuals with the AA genotype had a 2.7-fold higher risk for developing JIA, thus revealing that patients were more susceptible to JIA. Furthermore, we detected a significant negative association between JIA and the G allele at the −1082 position (OR: 0.6; *P* <0.05) indicating that this association could represent a protective effect against JIA. However, we did not find any significant association between the genotype or allele frequencies and susceptibility to JIA for the −819 and −592 positions of the IL-10 promoter regions.

Several recent studies revealed that polymorphisms of various cytokine genes may alter gene expression, and lead to the development of JIA or change its clinical manifestations [22–27]. An earlier study by Huizinga et al. [26] reported a lower IL-10 mRNA content in synovial biopsies from adult patients with destructive joint disease compared to those with an inflammatory but non-destructive joint disease. This research revealed that IL-10 promoter genotype could predict joint destruction. The authors added that the presence of the allele associated with less joint destruction was correlated with higher IL-10 production, suggesting a protective role of IL-10 in RA. Crawley et al. [27] demonstrated that patients with juvenile RA with more than four joints affected were more likely to have a particular IL‐10 genotype different from those with fewer than four joints affected.

Our study confirm and extend these findings as a significant positive association was evident between the IL-10 (-1082) AA genotype and susceptibility to polyarticular JIA among studied patients (OR: 4.3; *P* <0.01). In addition, we observed that JIA patients with the AA genotype had significantly lower serum IL-10 levels compared to those with the AG and GG genotypes. Furthermore, we found that the A allele at the same position was associated with lower serum IL-10 levels, meanwhile the G allele was correlated with higher IL-10 levels among studied JIA patients. These results were concordant with those of Stanilova et al. [28] who reported that the A to G switch at position −1082of the IL-10 gene was associated with increased IL-10 production, and the AA, AG, and GG genotypes being associated with low, intermediate, and high interleukin −10 production, respectively.

In accordance with our results, Crawley et al. [27] studied IL10 gene SNPs (at −1082A/G, −819C/T, and −592A/C) on genomic DNAs of 444 Caucasian children with JIA, compared to 274 healthy control children. The authors found an increased frequency of the ATA haplotype in patients with extended OJIA. They also reported that there is a transcriptional variation in IL-10 production among different genotypes. Their findings confirm an association between the “low” IL-10–producing haplotype and disease severity in children with oligoarticular-onset JIA that extends to polyarthritis.

In the current study, no significant association was evident between Il-10 gene variants and susceptibility to systemic onset JIA. By contrast, a study performed by Fife et al. [29] from the UK identified increased prevalence of the IL10-1082 A allele, associated with low IL10 production, in patients with systemic onset JIA. Omoyinmi et al. [30] confirmed that two *IL-10* genetic variants (rs1878672 and rs1800896) showing some evidence of association with systemic onset JIA in Caucasian population. It may be assumed that interaction between IL10 and other cytokines in antigen presentation and T cell polarization pathways could determine the phenotype of the disease. Certainly, the small number of studies concerning cytokine gene polymorphisms, particularly in Caucasian population, makes it difficult to express explicitly the hypotheses or concepts.

Experimental models of collagen-induced arthritis revealed that recombinant IL-10 decreases the incidence, delays the onset, and reduces the severity of arthritis [31]. An earlier study by Joosten et al. [32] confirmed that the addition of exogenous IL‐10 in these models also had a clear chondroprotective effect. Moreover, synovial tissue cultures indicated that IL‐10 functions by inhibition of the production of IL‐1, TNF-α, and matrix metalloproteinases and induces the production of tissue inhibitor of metalloproteinases 1 (TIMP 1). These metalloproteinases are responsible for cartilage degradation. Therefore, a lack of IL-10 can lead to increased expression of metalloproteinases and reduced TIMP 1 expression, allowing joint destruction [33].

Of note, we observed that patients with polyarticular JIA did show significantly lower serum IL-10 levels compared to patients with systemic JIA and those with oligoarticular subtypes. Our results were different from those of Prahalad et al. [10] who reported that IL10 was significantly elevated in polyarticular as well as systemic subtypes of JIA. However, we could not find any significant difference between JIA cases and controls as regards serum IL-10 levels as they did. The systemic concentrations of IL10 are mainly regulated at the level of expression, because IL10 is rapidly cleared from the plasma as it has a plasma half-life of only a few hours [18]. Therefore, this discrepancy may be attributable to methodological issues, such as test specimen (serum versus plasma) and assay methodology or to the differences in the size of the cohorts. However, more studies on cytokine profiles in patients with JIA are needed for a definite conclusion.

On the other hand, no significant association was evident between different clinical subtypes of JIA or serum IL-10 levels and the genotype or allele frequencies for the −819 and −592 positions of the IL-10 promoter regions.

A few studies in the literature concerned the association of human IL-10 gene polymorphisms with JIA and the susceptibility to JIA [27, 29, 30, 34]. To our knowledge, ours is the first such study performed in an Egyptian population. However, the small sample size was one of our limitations in this study; we suggest that multicenter approaches may be necessary to attain larger sample size. Cytokine profile was not measured during remission as all studied JIA patients had an active disease. Therefore, the patterns of cytokine expression could not be determined. Another limitation in our study was that IL‐10 production may be altered in many children with arthritis because of the treatment they receive, which includes both glucocorticoids and MTX; these are both known to affect IL‐10 production.

## Conclusion

We demonstrate for the first time, to the best of our knowledge, that the presence of an A allele or AA gene variant at the −1082 position of the promoter region of the interleukin-10 gene may constitute risk factors for developing JIA in Egyptian children and adolescents. Moreover, we observed a significant positive association between the IL10 –1082 AA gene variant and susceptibility to polyarticular JIA.

Further studies and more genetic information on ethnicities from different parts of the world will provide an additional understanding of the possible role of cytokine gene polymorphisms in JIA aiming to improve diagnosis, assess severity, and thus seek for new therapeutic modalities of such disease.

## Abbreviations

ARMS: Amplification refractory mutation system
CI: Confidence interval
CRP: C‐reactive protein
ELISA: Enzyme-linked immunosorbent assay
ESR: Erythrocyte sedimentation rate
IL-10: Interleukin-10
IL-10R: Interleukin-10 receptor
IL-6: Interleukin-6
JIA: Juvenile idiopathic arthritis
OR: Odds ratio
PCR: Polymerase chain reaction
SNPs: Single nucleotide polymorphisms
TNF-α: tumor necrosis factor-α
